# C_15_-bacillomycin D produced by *Bacillus amyloliquefaciens* 4-9-2 suppress *Fusarium graminearum* infection and mycotoxin biosynthesis

**DOI:** 10.3389/fmicb.2025.1599452

**Published:** 2025-06-16

**Authors:** Zhongliang Liu, Yijia Luo, Rongxin Lin, Chengming Li, Hanjun Zhao, Haqmal Mohammad Aman, Muhammad Asif Wisal, Huifeng Dong, Dingkuo Liu, Xiaona Yu, Lingcong Kong, Hongxia Ma

**Affiliations:** ^1^College of Life Science, Jilin Agricultural University, Changchun, China; ^2^College of Animal Science and Technology, Jilin Agricultural University, Changchun, China; ^3^Tianjin Key Laboratory of Biological Feed Additive Enterprise, S&E Burgeoning Biotechnology (Tianjin) Co., Ltd., Tianjin, China; ^4^College of Nursing, He University, Shenyang, China

**Keywords:** biological control, lipopeptides, *F. graminearum*, antifungal activity, mycotoxin

## Abstract

**Introduction:**

*Fusarium graminearum* threatens global food security through crop diseases and mycotoxin contamination, presenting significant challenges in controlling this toxigenic pathogen.

**Methods:**

Soil bacteria were isolated and screened for antagonism using plate confrontation. Active strain 4-9-2 was identified by 16S rDNA and whole-genome sequencing. Antifungal metabolites were characterized via AntiSMASH, HPLC, and ESI-IT-TOF/MS. MIC and IC₅₀ against *F. graminearum* spores/hyphae were determined. Biocontrol efficacy was tested on maize kernels, measuring infection suppression and mycotoxin reduction. Compound stability was assessed under varying temperatures (25–100°C), pH (2–12), metal ions, and enzymes. Mechanisms were investigated through microscopy, membrane permeability, ROS, and membrane potential assays.

**Results:**

Strain 4-9-2 (*Bacillus amyloliquefaciens*) showed potent antifungal activity. Its genome (3,957,046 bp, GC 46.5%) harbored 12 BGCs. The metabolite was identified as C_15_-bacillomycin D, inhibiting *F. graminearum* at MIC 64 μg/mL and IC_50_ 26.10 μg/mL. It suppressed maize kernel infection and reduced deoxynivalenol (DON) and zearalenone (ZEN) levels. Bacillomycin D maintained activity across tested temperatures, pH, and stressors. It disrupted membrane integrity, causing morphological defects, increased permeability, ROS accumulation, and membrane depolarization.

**Discussion:**

Bacillomycin D from *B. amyloliquefaciens* 4-9-2 is a promising biocontrol agent against *F. graminearum*, combining potent antifungal activity, mycotoxin reduction, environmental resilience, and membrane-targeting action.

## 1 Introduction

*Fusarium graminearum* is a highly destructive fungal pathogen widely distributed across the globe, particularly in warm and humid climates. This pathogen not only severely affects important cereal crops such as wheat, maize, and barley, but also produces several harmful mycotoxins, most notably deoxynivalenol (DON) and zearalenone (ZEN) (Thielecke and Nugent, 2018). Research has shown that DON accumulation can lead to acute symptoms of toxicity, such as vomiting and diarrhea, and long-term exposure is associated with immune suppression and neurological damage (Murtaza et al., 2024). ZEN has adverse effects on the growth and reproductive systems of livestock (Liu and Applegate, 2020). These toxins can accumulate in contaminated crops, directly impacting food safety and human health. It is estimated that the global food losses caused by *F. graminearum* amount to billions of dollars annually, especially in major agricultural regions such as Asia, North America, and Europe (Mesterhazy, 2024). Effective control methods are urgently needed to address this issue.

Currently, the primary method for controlling *F. graminearum* is the use of chemical pesticides (Anderson et al., 2020; Avenot and Michailides, 2010; Bolanos-Carriel et al., 2020). However, prolonged reliance on chemical agents not only leads to the development of resistance but also causes environmental pollution and harm to non-target organisms (Zhao H. et al., 2022). Moreover, a recent study indicated that chemical pesticides can enhance the synthesis of mycotoxins (Duan et al., 2020). Therefore, it is necessary to explore alternatives or supplementary methods to chemical fungicides for effective control of Fusarium in wheat, ensuring food safety and environmental quality. With the increase in consumer demand for safe, healthy, and environmentally friendly food, research and application of more natural and pollution-free control measures have been promoted. Among them environment- and ecosystem-friendly biocontrol agents have garnered increasing attention worldwide (Ayaz et al., 2023).

Biological control has become an effective and environmentally friendly method for managing *F. graminearum* contamination, gaining increasing attention in recent years (Legrand et al., 2017). Many microorganisms, particularly species of the genus *Bacillus* spp., possess excellent abilities to inhibit fungal growth. These bacteria not only compete with molds for resources and space but also produce various antifungal substances, such as iturins, fengycins and surfactins, among which iturins has been widely studied due to its good antifungal activity (Gu et al., 2017; Yaraguppi et al., 2023). The iturin family includes a variety of members, mainly including iturin A, iturin C, iturin D, iturin E, iturin F, iturin W, bacillomycin D, bacillomycin F, bacillomycin L, mixirins (Wan et al., 2022; Zhou et al., 2020). Studies have already demonstrated their effectiveness in combating *F. graminearum* contamination. For example, research has shown that *B. amyloliquefaciens* FZB42 demonstrates significant antifungal activity toward *F. graminearum*, and its ability to produce bacillomycin D is key in suppressing the growth of *F. graminearum* (Gu et al., 2017). Other studies have found that iturin A isolated from *B. amyloliquefaciens* JCK-12 synergistically and significantly inhibits the spore germination of *F. graminearum* (Gong et al., 2015). Therefore, the ongoing search for functional strains with excellent antifungal properties against *F. graminearum* may provide higher-quality and more suitable resources for developing novel biopesticides and biocontrol agents.

This study screened out an antagonistic strain from soil microorganisms that exhibited significant inhibitory effects on *F. graminearum*, identified as *B. amyloliquefaciens* 4-9-2. After cultivation, a itrurin-like antifungal lipopeptide was purified and characterized from its cell-free supernatant. By molecular weight comparison, it was identified as C_15_-bacillomycin D. Subsequently, the inhibitory activity of bacillomycin D against *F. graminearum* was tested, and the stability of its antifungal activity was studied. The mechanism of action of bacillomycin D against *F. graminearum* was further explored, including observations of hyphal morphology, detection of cell membrane permeability, and changes in reactive oxygen species and membrane potential.

## 2 Materials and methods

### 2.1 Isolation of antagonistic bacteria against *F. graminearum*

The *F. graminearum* used in the laboratory is the standard strain, purchased from the China Center for Type Culture Collection (CTCC), with the number AS3.3488. Soil samples were collected from Jilin Province, China, and transferred to the laboratory within 24 h for bacterial isolation. The soil samples (1 g) were suspended in 99 mL of sterile distilled water (SDW) in a 250 mL conical flask and thoroughly mixed for 1 h at 25°C, using a magnetic stirrer. Subsequently, the soil suspension was serially diluted (10^−1^, 10^−2^, and 10^−3^) with SDW and 100 μL of each dilution was inoculated onto LB agar (Luria-Bertani Agar) medium. The plates were incubated at 30°C for 24–48 h, and newly formed colonies on the plates were picked, streaked to purify the strains, and repeated three times.

### 2.2 Determination of antagonistic bacteria inhibition rate against *F. graminearum*

The antagonistic activity of the isolated strains against *F. graminearum* was detected using the plate confrontation method. Briefly, the test strains were inoculated into LB medium and incubated at 37°C with shaking for 12 h. Then, a mycelial plug (6 mm in diameter) of *F. graminearum* was cut from a 3-day-old PDA culture and placed in the center of the PDA (Potato Dextrose Agar) plate. The test bacteria were dropped onto sterile filter paper disks (5 μL) placed 2 cm away from the center. Plates inoculated with only *F. graminearum* mycelial disks served as controls. Each bacterium was tested on three replicate plates. The plates were incubated at 28°C for 3–5 days, or until the mycelium of *F. graminearum* covered the entire plate in the control. The colony diameters of *F. graminearum* in the treatment and control groups were measured, and the inhibition rate of the strains was calculated. The calculation method is shown in Equation 1.

(1) Inhibition rate=[(C−T)/C]×100%

Among them, C was the average colony diameter of the control group. T was the average colony diameter of the treatment group.

### 2.3 Identification of antagonistic bacteria against *F. graminearum*

Genomic DNA was extracted from the bacterial strain using a bacterial genomic DNA extraction kit, and its 16S rDNA fragment was amplified by PCR. The sequence of the forward primer 27F used for amplifying the 16S rDNA fragment was 5′-AGAGTTTGATCCTGGCTCAG-3′, and the sequence of the reverse primer 1492R was 5′-GGTTACCTTGTTACGACTT-3′. The 16S rDNA fragment was sequenced, and the sequence was compared to highly similar sequences downloaded from the EzBioCloud database. Multiple sequence alignments were performed using MAFFT, gaps were processed with Gblocks, nucleotide substitution model parameters were generated with MrModelTest, and a phylogenetic tree was constructed using MrBayes.

### 2.4 Whole-genome sequencing

Genomic DNA was quantified using the Qubit 4 Fluorometer (Life Technologies, United States), and libraries were prepared with the SQK-LSK109 Ligation Sequencing Kit (Oxford Nanopore Technologies, United Kingdom). The experimental process followed the standard protocol provided by Oxford Nanopore Technologies (ONT), including sample quality testing, library construction, library quality testing, and library sequencing. For genome assembly, the filtered reads were assembled using Canu v1.5, followed by circularization of the assembly with Circlator v1.5.5. For genome annotation, coding genes were predicted using Prodigal v2.6.3.

### 2.5 The genomic bioinformatics analyses

The GenBlastA v1.0.4 program was used to scan the whole genome after masking predicted functional genes. Gene prediction was performed using the Rapid Annotation using Subsystem Technology (RAST) SEED viewer^1^ (Brettin et al., 2015). AntiSMASH v6.0.1 was applied to identify secondary metabolite biosynthesis gene clusters (Blin et al., 2021).

### 2.6 The preparation of antifungal lipopeptides

*Bacillus amyloliquefaciens* 4-9-2 was inoculated into LB medium and cultured at 37°C with shaking at 180 rpm for 72 h. After cultivation, the fermentation broth was centrifuged at 12,000 rpm for 10 min, and the cell-free supernatant (CFS) was then collected. The CFS was treated with 6 M HCl to adjust the pH to 2 and stored overnight at 4°C to allow complete precipitation of the lipopeptides (Yakimov et al., 1995). The precipitated material was collected by centrifugation (12,000 rpm, 30 min, 4°C) and extracted at least twice with methanol to obtain a lipopeptide crude extract.

### 2.7 The purification of antifungal lipopeptides

The lipopeptides were further purified using preparative HPLC (Waters 2,489, Waters Technology, Shanghai, China), equipped with a 5 μm C18 column (Waters Technology, Shanghai, China), for the preparation and separation of the antagonistic substances. The fractions exhibiting antifungal activity were dried with a nitrogen blower. The sample was then dissolved in chromatographic methanol, filtered through a 0.22 μm organic phase filter, and placed in a vial for storage. The injection volume was 1 mL. Mobile phase A consisted of ultrapure water (containing 0.1% trifluoroacetic acid), and mobile phase B was chromatographic-grade acetonitrile (containing 0.1% trifluoroacetic acid), with ultrasonication for 20 min to remove bubbles. The elution flow rate was set at 2 mL/min for both mobile phases A and B. The elution conditions were programmed to increase the acetonitrile concentration from 5 to 100% over 0–60 min. The wavelength was set to 214 nm, and a single peak was observed and collected.

### 2.8 ESI-IT-TOF/MS analysis of antimicrobial compounds

ESI-IT-TOF/MS analysis was performed using a Surveyor-LCQ DECA XP Plus (Thermo Finnigan, Thermo Electron Corporation, San Jose, CA, United States). The electrospray source was operated at a capillary voltage of 32 V, a spray voltage of 5 kV, and a capillary temperature of 27°C.

### 2.9 Determination of minimum inhibitory concentration (MIC) and spore germination inhibition rate

The antifungal effect of bacillomycin D on *F. graminearum* was determined using a broth microdilution method (Alastruey-Izquierdo et al., 2008; Gimenez et al., 2021). Different concentrations of bacillomycin D were prepared in 90 μL culture medium using a 96-well flat-bottom microculture plat double dilution method. The final concentrations of bacillomycin D in each sample well were 512 μg/mL, 256 μg/mL, 128 μg/mL, 64 μg/mL, 32 μg/mL, 16 μg/mL, 8 μg/mL, and 4 μg/mL, with each concentration tested in duplicate. Subsequently, 10 μL of spore suspension was added to each well, resulting in a final spore concentration of 1 × 10^5 CFU/mL. Methanol was added as a negative control, and an equal volume of PDB (Potato Dextrose Broth) medium was added as a blank control. After incubation at 28°C for 48 h, the OD600 values in each well were measured using a Spectrophotometer 1,500 (Thermo Fisher Scientific, United States) to assess fungal growth.

The germination rate of conidia was determined using the same microdilution broth dilution method and calculated as the ratio of germinated conidia to the total number of spores. All treatments were incubated at 28°C, and the number of germinated spores was counted using an inverted microscope (Nikon Diphot, Japan) after 12 h. The minimum inhibitory concentration (MIC) was defined as the concentration of the antifungal compound that completely inhibits fungal growth or conidial germination.

### 2.10 Effect of bacillomycin D on mycelial growth

To assess the inhibitory activity against mycelial growth, the bacillomycin D was dissolved in methanol to prepare solutions at various concentrations. Next, 0.2 mL of the bacillomycin D solution was added to 14.8 mL of PDA medium, which had been cooled to 40–50°C, and the mixture was poured into 60 mm petri dishes, with 5 mL per plate. The final concentration of lipopeptide were 80 μg/mL, 40 μg/mL, 20 μg/mL, 10 μg/mL, 5 μg/mL. The control group received methanol instead of the bacillomycin D solution. A 5 mm mycelial disk was inoculated at the center of each plate in both the treatment and control groups, and the plates were then incubated at 28°C. When the mycelial colonies of the control group grew the edge of the petri dish (approximately 96 h later), the colony diameter of each treatment and control group was measured using the cross method. The rate of mycelial growth inhibition was calculated using the following formula, and the 50% inhibitory concentration (IC_50_) was obtained by employing GraphPad Prism 9.5 software. The calculation method is shown in Equation 2.

(2) $$\text{Growth inhibition rate}=[\begin{array}{l} (\begin{array}{l} \text{mycelial colony diameter}\;\\ \text{of the control group}-\\ \text{mycelial colony diameter}\;\\ \text{of the bacillomycin}\;D-\\ \text{treated group} \end{array})/\\ \text{mycelial colony}\;\\ \text{diameter}\;\\ \text{of the control group} \end{array}]\times 100\%$$

### 2.11 Effect of bacillomycin D on *F. graminearum* infection corn

To further evaluate the biocontrol potential of bacillomycin D in practical use, corn seeds were randomly divided into two groups, with 10 seeds in each group. One group was soaked in a bacillomycin D solution at 64 μg/mL, while the other group was soaked in a methanol solution as a control. After the surface samples of the two groups were dried, the spores of *F. graminearum* (final spore concentration 1 × 10^5 CFU/mL) were evenly sprayed, and the samples were then placed in an incubator at 28°C for continuous observation, and the growth and contamination of *F. graminearum* in each group were recorded. The toxin content in each treatment group was measured using DON and ZEN test kits (Purebon Biotechnology Co., Ltd., Qingdao, China).

### 2.12 Determination of bacillomycin D stability

Stability of bacillomycin D against *F. graminearum* was assessed using filter paper method (Li et al., 2022). Briefly, a 6 mm-diameter sterilized disk was placed on PDA plates, followed by adding 7 μL of bacillomycin D (2.5 mg/mL) solution or solvent onto the disk. After drying, 5 μL (1 × 10^5 CFU/mL) of *F. graminearum* spore suspension was added to the disk. The plates were incubated at 28°C for 24 h, and colony diameters were measured.

The pH stability of bacillomycin D was tested by adjusting the pH of the peptide solution to values ranging from 2 to14 and incubating it at room temperature. The thermal stability of bacillomycin D was tested by soaking them in water at 50°C and 100°C for 30 min, with room temperature-treated (25°C) samples serving as the control. The stability of bacillomycin D against metal ions was tested by mixing 10 nM of different metal ions (Na^+^, K^+^, Ca^2+^, Mg^2+^, Cu^2+^, Fe^2+^, Fe^3+^) with the peptide solution and incubating it at room temperature. The stability of bacillomycin D against proteolytic enzymes was tested by treating them with different proteases (such as proteinase K, trypsin, papain, and pepsin, purchased from Sangon Biotechnology, Shanghai, China) for 1 h, followed by a incubation at 37°C. The concentration of the enzyme was 5 mg/mL, and the reaction was terminated by a 10-min water bath at 100°C, as described previously. The researchers used the bacillomycin D without any treatment as a control to compare the differences in antimicrobial effects of bacillomycin D under different treatment conditions. Statistical analysis data were expressed as mean ± standard deviation (SD) from three independent experiments.

### 2.13 Microscopic analysis

*Fusarium graminearum* was inoculated into tubes containing PDB medium and cultured at 28°C with shaking (100 rpm) for 12 h. Afterward, the bacillomycin D solution was added at a final concentration of 16 and 32 μg/mL, and the fungus was cultured for an additional 12 h. A control group was set up by adding the same amount of PDB to another tube containing *F. graminearum*. After completing the culture, the microscopic cell structure of fungal hyphae was observed using scanning electron microscopy (SEM) and transmission electron microscopy (TEM).

For SEM, the fungal hyphae were fixed in 2.5% glutaraldehyde (prepared in 0.1 M sodium phosphate buffer) at 4°C for 24 h, rinsed three times with phosphate buffer (0.02 M), and then fixed with 2% osmium tetraoxide for 2 h at 20°C. The samples were subsequently dehydrated in a graded series of ethanol concentrations (30, 50, 75, and 95%) for 10 min at each concentration, followed by CO_2_ critical point drying. The samples were then sputter-coated with a gold–palladium alloy using a Nanotech sputter coating apparatus (ES-2030 HITACHI, Japan). The samples were stored in a desiccator until examined with a scanning electron microscope (Philips, SEM-505, Holland) operated at 30 kV.

For TEM, a procedure similar to SEM was followed until the fungal hyphae were fully dehydrated. After dehydration, the samples were embedded in Epon810 resin, and ultrathin sections (80 nm) were cut at room temperature using an ultra-microtome RMC-MT7000 with a diamond knife. After the tissue was mounted on a copper grid, post-staining was performed with uranyl acetate for 30 min, followed by lead citrate for 20 min. The samples were stored in desiccators until examined with a TEM (HITACHI, H-600, Japan) operated at 120 kV.

### 2.14 Membrane integrity testing

*Fusarium graminearum* was treated with bacillomycin D for 1 h, and then the treated and untreated mycelial suspensions were mixed with 50 μmol/L Sytox Green and 1 μmol/L DAPI. Sytox Green is a high-affinity nucleic acid stain that can easily penetrate cells when the cell membrane was damaged. DAPI is a fluorescent dye that can penetrate the cell membrane and bind to double-stranded DNA in the nucleus as a marker. After staining in the dark for 5 min, the dye was washed off and the hyphal suspension was resuspended in physiological saline. Fluorescence values of Sytox Green (488 nm/525 nm) and DAPI (365 nm/454 nm) were measured using a fluorescence spectrophotometer. A small amount of hyphae was then collected to prepare slides, and the effect of bacillomycin D treatment on hyphal cell membrane permeability was observed under a fluorescence microscope with an excitation wavelength of 465–495 nm.

### 2.15 The reactive oxygen species (ROS) detection

The endogenous ROS levels of *F. graminearum* were detected using fluorescence labeling technology, with the fluorescent dye DCFH-DA serving as the ROS indicator. Mycelia treated with bacillomycin D as previously described were collected. Mycelia treated with Rousp were used as a positive control, and methanol was used as a negative control. DCFH-DA was added to the collected mycelium suspension at a final concentration of 10 μM and incubated at 37°C for 30 min. After incubation, fungal cells were centrifuged at 12,000 r/min for 5 min and washed twice with PBS. The pellets were then resuspended in 0.5 mL PBS. The fluorescence values of each treatment group were measured using a F4500 fluorescence spectrophotometer (Thermo Fisher Scientific, Massachusetts, United States). The excitation and emission wavelengths were set at 485 nm and 525 nm, respectively.

### 2.16 The membrane potential detection

The impact of bacillomycin D on the membrane potential of *F. graminearum* was detected using the membrane potential-sensitive fluorescent dye DiSC3(5). Mycelia treated with lipopeptide were collected. Methanol was used as a negative control, while 0.1% Triton X-100 served as a positive control. The procedure involves the addition of DiSC3(5) to a final concentration of mycelial suspension 0.4 μM, followed by incubation at 37°C for 1 h. Changes in fluorescence intensity were measured using an F4500 fluorescence spectrophotometer (Hitachi Tokyo, Japan). The excitation and emission wavelengths were set at 622 nm and 675 nm, respectively.

### 2.17 Statistical analysis

Statistical analysis data are expressed as mean ± standard deviation (SD) from three independent experiments. Statistical significance was determined using one-way ANOVA in GraphPad Prism 9.5. A *p*-value < 0.05 was considered significant.

## 3 Results

### 3.1 The screening of antagonistic strains against *F. graminearum*

**Figure 1:**
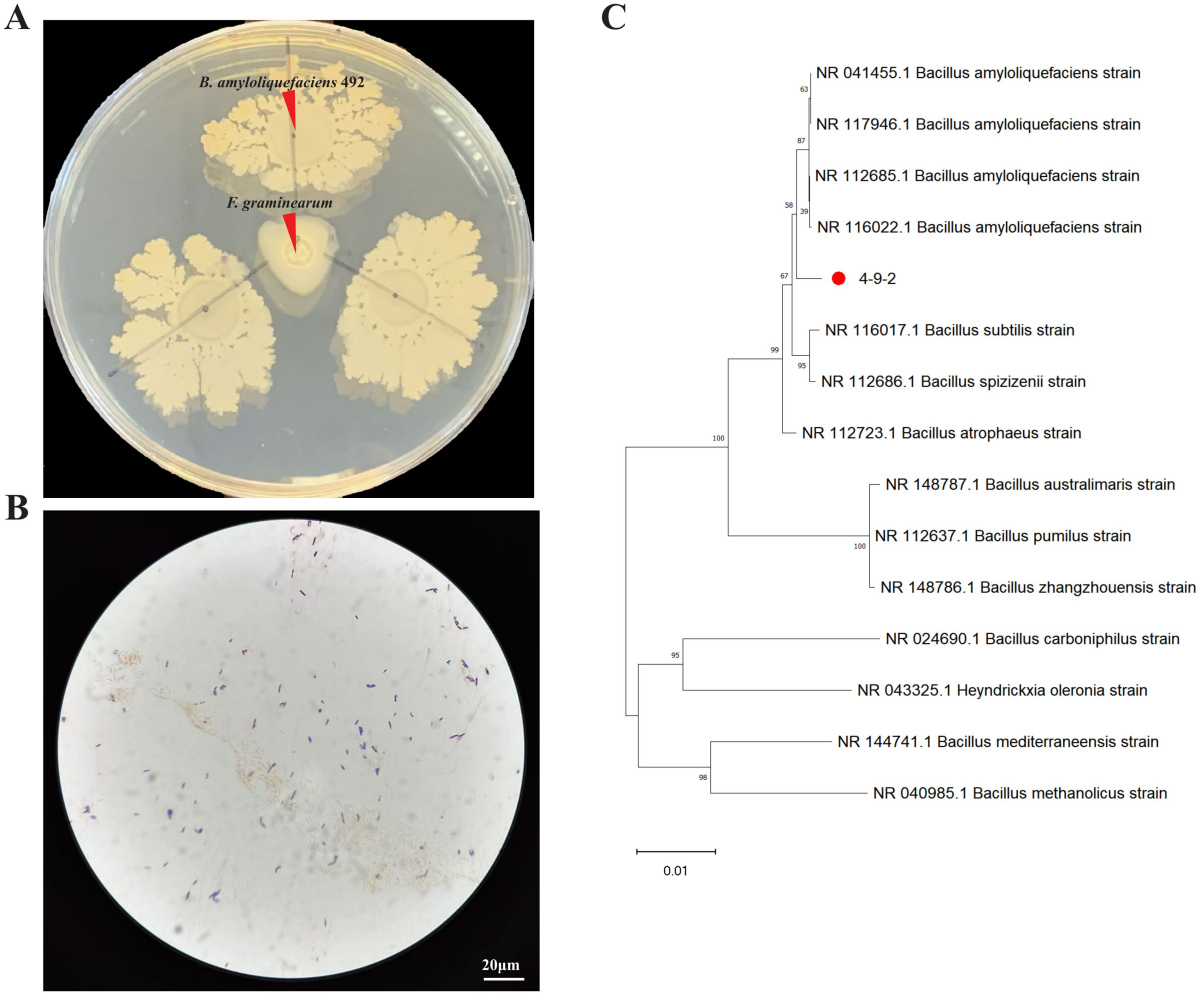
Screening and identification of antagonistic bacteria against *Fusarium graminearum*. **(A,B)** The antifungal effect and Gram staining of strain 4-9-2 against *F. graminearum*. **(C)** A phylogenetic tree of strain 4-9-2 was constructed using Bayesian method based on 16s rDNA sequence.

After screening, strain 4-9-2 demonstrated remarkable antagonistic activity against *F. graminearum*. The colony of strain 4-9-2 is milky white in color, with a raised and wrinkled surface, and its margins were irregular. Gram staining revealed that the cells were Gram-positive and appeared as short rods (Figures 1A,B).

### 3.2 The identification of antagonistic strains 4-9-2

The 16S rDNA gene sequence of strain 4-9-2 was analyzed using Blast software on the NCBI website. Strain 4-9-2 showed more than 99% homology with several Bacillus strains in the NCBI database, as illustrated in the phylogenetic tree (Figure 1C). Based on its morphological characteristics and molecular biological identification, the antagonist strain 4-9-2 was identified as *B. amyloliquefaciens*. Therefore, it was designated as *B. amyloliquefaciens* 4-9-2.

### 3.3 Genomic features and secondary metabolic related genes of *B. amyloliquefaciens* 4-9-2

**Figure 2:**
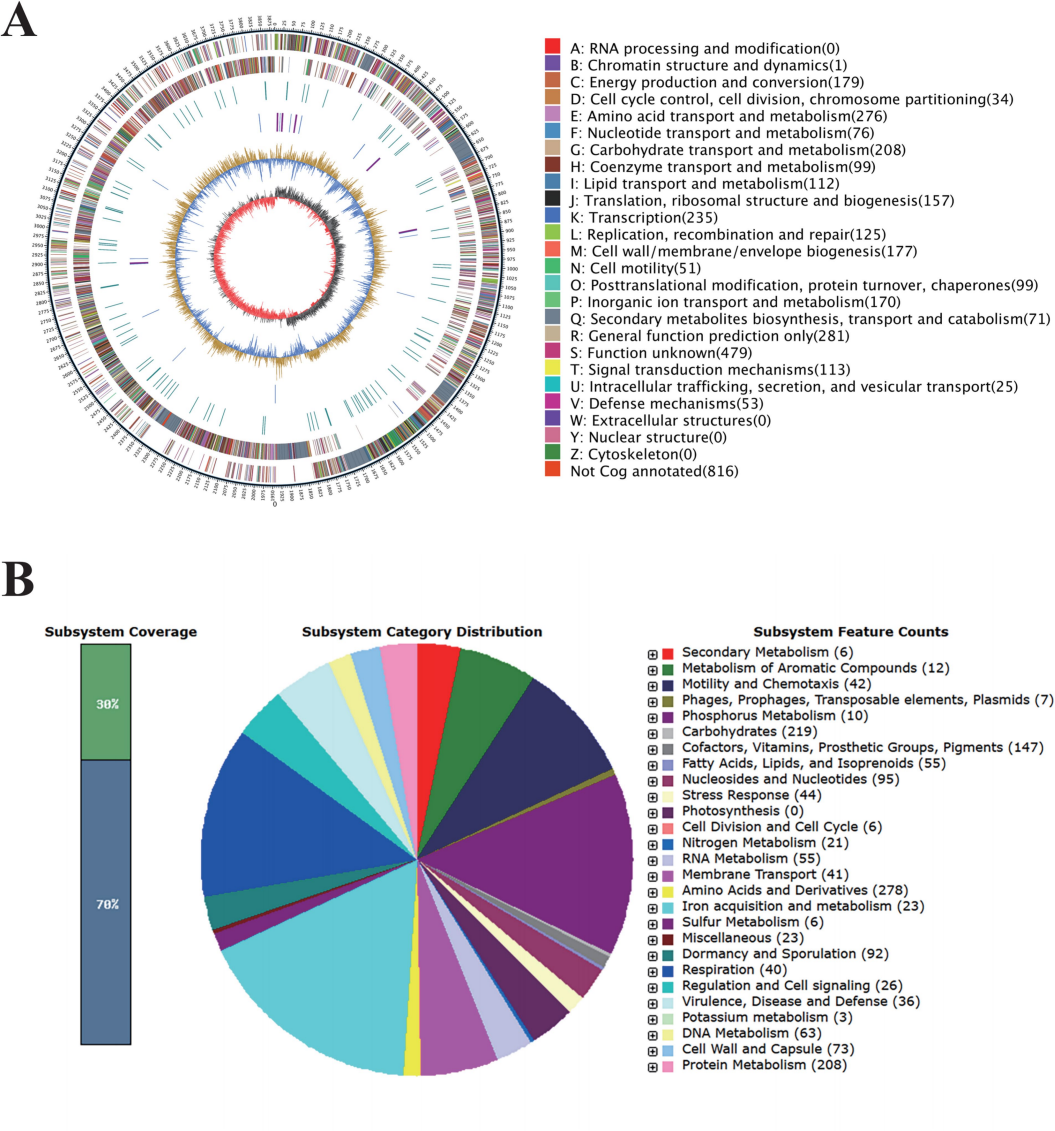
Genomic Features of *Bacillus amyloliquefaciens* 4-9-2. **(A)** Circle diagram of the assembled genome. **(B)** The complete genome sequence of *B. amyloliquefaciens* 4-9-2 was annotated based on RAST serve.

To further investigate the biological control potential of *B. amyloliquefaciens* 4-9-2, whole genome sequencing analysis was conducted. The complete genome sequence of *B. amyloliquefaciens* 4-9-2 consists of a circular chromosome of 3,957,046 base pairs (bp). This chromosome contains 3,716 coding sequences (CDS), with an average length of 945 bp and a G + C content of 46.5%. Additionally, the genome includes 27 ribosomal RNA (rRNA) genes, 84 transfer RNA (tRNA) genes, and 9 genomic islands, each averaging 24,022.67 bp in length. Of the 3,716 CDS, 3,709 have functional annotations, while 7 remain hypothetical (Figure 2A).

The RAST server annotated 3,926 coding sequences and categorized them into subsystems, as illustrated in the pie chart in Figure 2B. Notably, 23 genes are associated with iron acquisition and metabolism, which could enhance the bacterium’s ability to thrive in nutrient-limited environments. Furthermore, 36 genes are linked to virulence, disease, and defense mechanisms, suggesting potential competitive advantages against other microorganisms. Six genes are involved in secondary metabolism, and 12 genes are dedicated to the metabolism of aromatic compounds, which may play roles in environmental adaptation. Additionally, 44 genes related to stress responses, including those for cold and heat shock, oxidative stress, osmotic stress, and detoxification, indicate a robust adaptability to environmental changes.

To explore the strain’s capacity for producing bioactive compounds, antiSMASH was employed to identify secondary metabolite biosynthesis genes clusters (BGCs). The BGC analysis revealed that the genome of *B. amyloliquefaciens* 4-9-2 is predicted to contain 12 biosynthetic gene clusters. Among these, macrolactin H, bacillaene, fengycin, difficidin, bacillibactin, and bacilysin exhibit 100% similarity to known gene clusters, while surfactin, myxovirescin A1, and Butirosin A/butirosin B show similarities of 78, 21, and 7%, respectively (as detailed in Table 1). These findings suggest that *B. amyloliquefaciens* 4-9-2 may synthesize lipopeptides that inhibit fungal pathogens, potentially offering a biological solution for controlling microbial contamination.

**Table 1:** Predicted secondary metabolism gene clusters identified by antiSMASH in *B. amyloliquefaciens* 4-9-2.

Gene clusters	Types	Genome locations	Most similar known clusters	Similarity
Cluster 1	NRPS	240,673–306,080	Surfactin	78%
Cluster 2	T1PKS, transAT-PKS	584,968–696,417	Myxovirescin A1	21%
Cluster 3	PKS-like	915,924–957,168	Butirosin A/butirosin B	7%
Cluster 4	Terpene	1,039,945–1,060,685		
Cluster 5	transAT-PKS	1,339,617–1,427,832	Macrolactin H	100%
Cluster 6	transAT-PKS, T3PKS, NRPS	1,646,486–1,756,576	Bacillaene	100%
Cluster 7	NRPS,transAT-PKS,	1,819,072–1,956,885	Fengycin	100%
Cluster 8	Terpene	1,979,830–2,001,713		
Cluster 9	T3PKS	2,052,136–2,093,236		
Cluster 10	transAT-PKS	2,251,743–2,357,925	Difficidin	100%
Cluster 11	NRP-metallophore, NRPS, RiPP-like	2,974,396–3,026,186	Bacillibactin	100%
Cluster 12	Other	3,546,822–3,588,240	Bacilysin	100%

Despite the promising genetic potential of *B. amyloliquefaciens* 4-9-2, there remains a paucity of research on the application of this bacterium in preventing and controlling *F. graminearum* contamination in grain. Therefore, this study investigates the efficacy of metabolites produced by *B. amyloliquefaciens* 4-9-2 in mitigating *F. graminearum* pollution, with a particular focus on their antifungal properties and potential as biocontrol agents.

### 3.4 Purification of the antifungal lipopeptide

**Figure 3:**
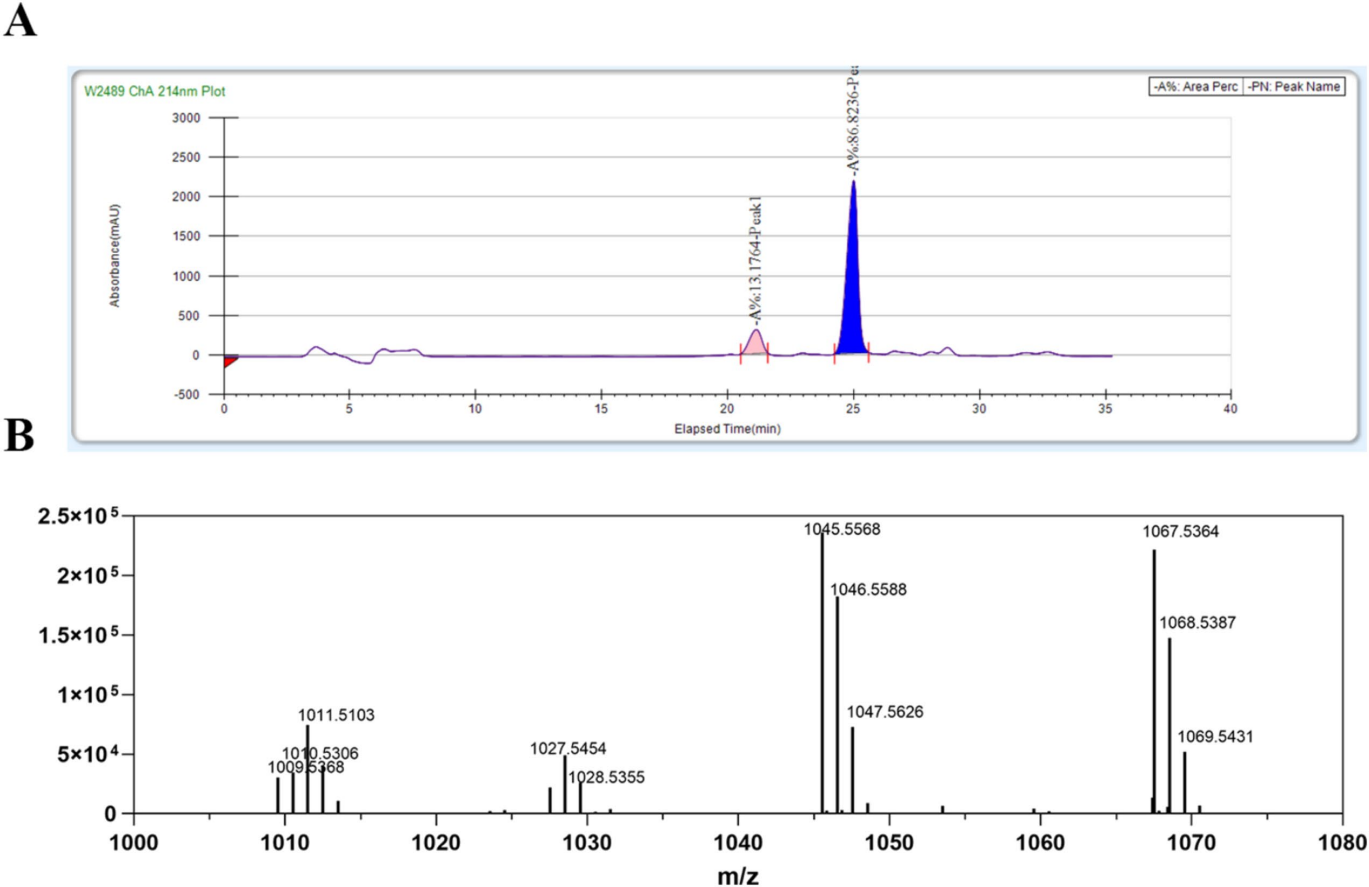
Purification and identification of lipopeptides (LPs) from *B. amyloliquefaciens* 4-9-2. **(A)** The purification process of LPs from *B. amyloliquefaciens* 4-9-2 via reversed-phase HPLC. The peak marked in pink is A1; the peak marked in blue is B1. **(B)** The ESI-IT-TOF/MS analysis results of the purified LPs.

The lipopeptide was precipitated from the fermentation broth using HCl and subsequently extracted with methanol. Following HPLC separation, two distinct active single peaks were identified and designated as A1 and B1 (Figure 3A).

### 3.5 Analysis of the antifungal lipopeptide

To further characterize the primary active compound, B1, we conducted ESI-IT-TOF/MS analysis. The positive ion mass spectrometry data revealed that the main ion peaks for the active substance B1 corresponded to bacillomycin D. Specifically, the molecular ion peaks [(M + H)^+^] for C_15_ bacillomycin D were observed at m/z 1,045.5, while the ion peaks [(M + Na)^+^] for the same compound were detected at m/z 1,067.5 (Figure 3B). These findings align with a previous study (Koumoutsi et al., 2004).

### 3.6 Antimicrobial activity and stability of bacillomycin D against *F. graminearum*

**Figure 4:**
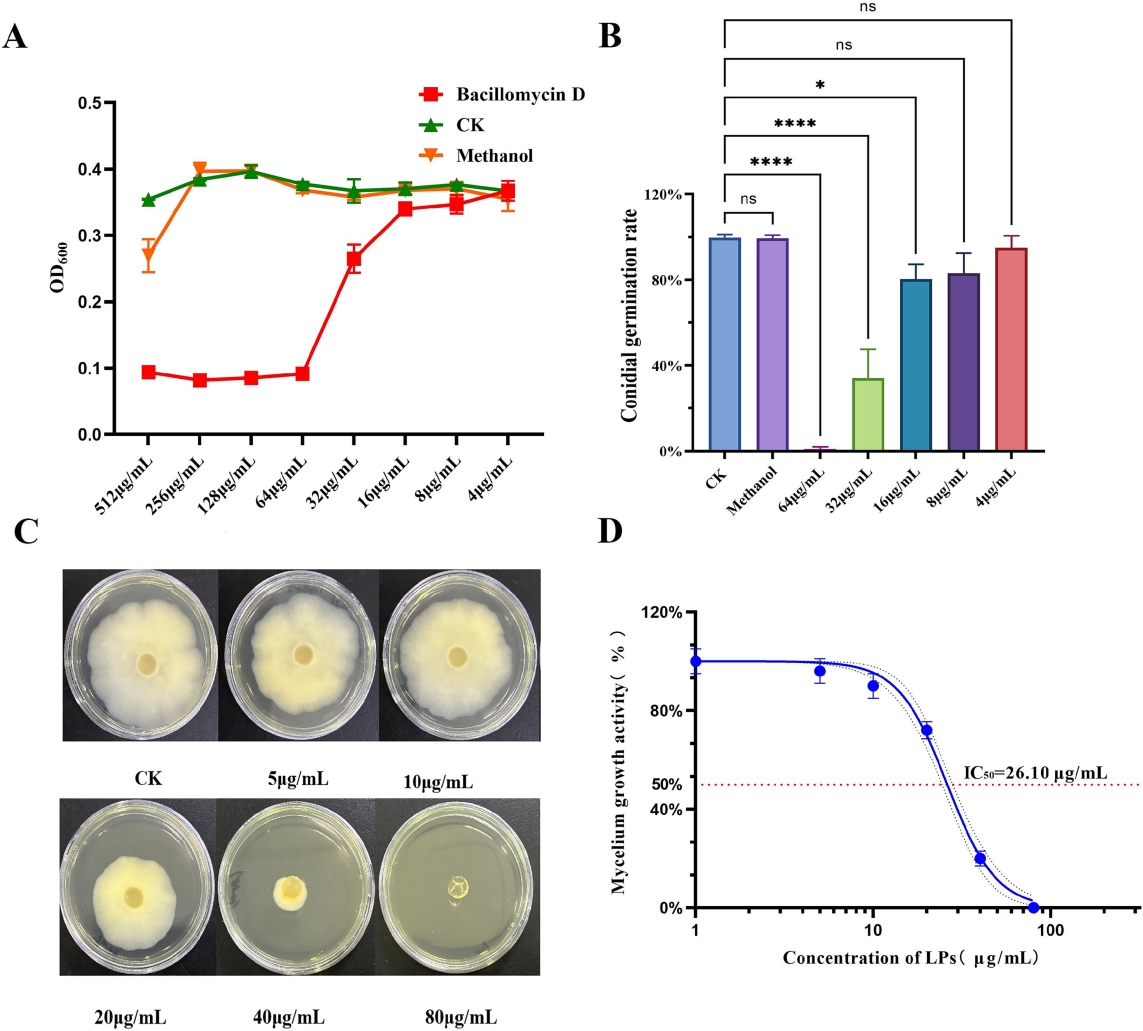
Determination of antifungal activities of bacillomycin D. **(A)** Determination of minimum inhibitory concentration (MIC) of bacillomycin D against *F. graminearum.* **(B)** Determination of inhibition rate of bacillomycin D on spore germination of *F. graminearum*. **(C,D)** Determination of the inhibitory ability of bacillomycin D on the mycelial growth of *F. graminearum*. CK represents the control group without the use of bacillomycin D treatment. *Significant differences were observed compared to the control group (*p* < 0.05). ****Super significant differences were observed compared to the control group (*p* < 0.0001).

In our study, the inhibitory activity of bacillomycin D against *F. graminearum* was evaluated. The results demonstrated that bacillomycin D at a concentration of 64 μg/mL completely inhibited spore germination (Figures 4A,B). Furthermore, bacillomycin D exhibited significant inhibitory activity against the mycelial growth of *F. graminearum*, with an IC_50_ of 26.10 μg/mL (Figures 4C,D).

**Figure 5:**
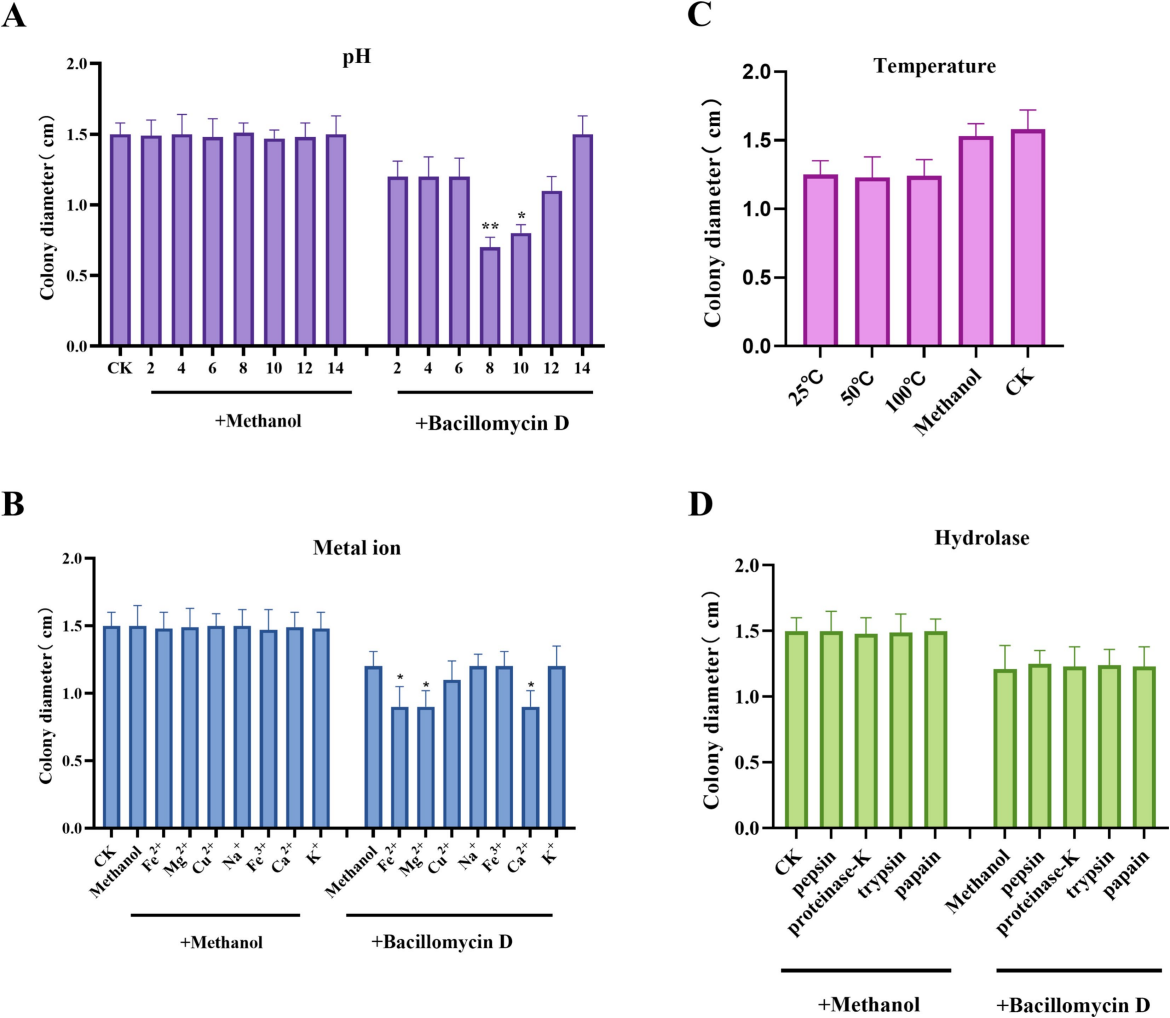
Environmental adaptability testing of bacillomycin D. **(A)** The effect of different pH levels on the antifungal activity of bacillomycin D Under normal conditions, the pH of the solution is close to 6. **(B)** The effect of different metal ion treatments on the antifungal activity of bacillomycin D. **(C)** The effect of different temperature treatments on the antifungal activity of bacillomycin D. Under normal conditions, the temperature of the solution is close to 25°C. **(D)** The effect of different hydrolytic enzyme treatments on the antifungal activity of bacillomycin D. CK represents the control group without the use of LPs treatment. *Significant differences were observed compared to the control group (*p* < 0.05). **Highly significant differences were observed compared to the control group (*p* < 0.01).

The stability of bacillomycin D under various conditions was also evaluated. Bacillomycin D maintained stable antibacterial activity after heating at 25–100°C, indicating good thermal stability. The activity of the bacillomycin D showed significant changes after incubation in solutions with pH values ranging from 2 to 14, with significant enhancement of antibacterial activity at pH 8 or 10, but complete loss of activity when the pH reached 14. Under other pH conditions, the activity remained stable (Figures 5A,C). Additionally, bacillomycin D retained stable antibacterial activity in the presence of different metal ions. Notably, divalent cations such as Fe^2+^, Mg^2+^, and Ca^2+^ were found to enhance their antifungal activity, which may be related to changes in hydrophobicity and molecular electrostatic interactions (Figure 5B). To further investigate the durability of bacillomycin D, their resistance to proteolytic degradation was tested. The results showed that bacillomycin D retained their activity after treatment with various proteases, including proteinase K, trypsin, pepsin, and papain (Figure 5D).

### 3.7 The biocontrol ability of bacillomycin D against *F. graminearum* pollution

**Figure 6:**
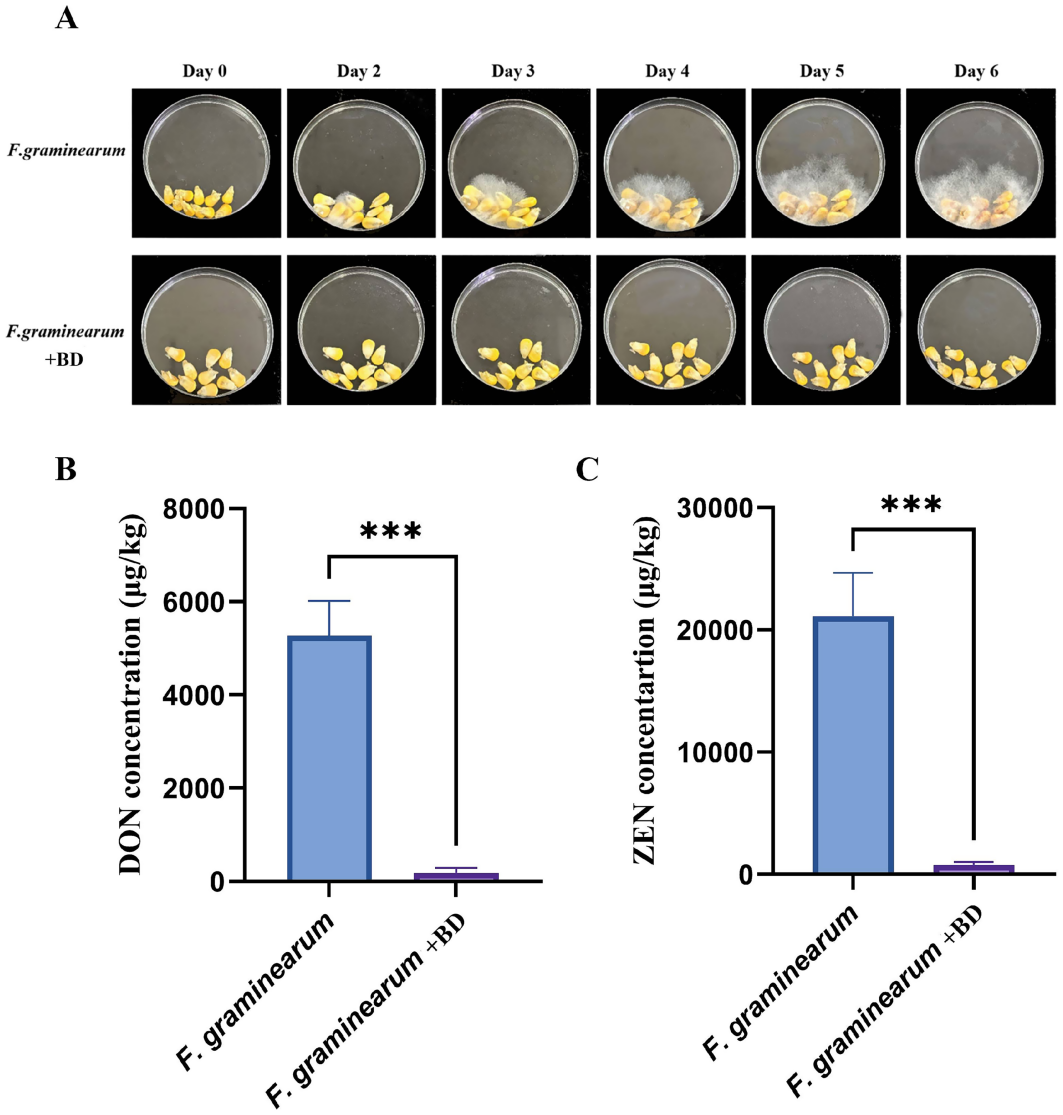
Determination of the biological control ability of bacillomycin D (BD) against *F. graminearum*. **(A)** The protective effect of bacillomycin D on corn seeds infected by *F. graminearum* was tested using a peptide concentration of 64 μg/mL. **(B,C)** Determination of DON and ZEN toxin content in two groups of corn seeds on the last day using bacillomycin D. ***Extremely significant differences were observed compared to the control group (*p* < 0.001).

To assess the practical effectiveness of bacillomycin D in controlling *F. graminearum*, infection assays were conducted. The results demonstrated that bacillomycin D significantly inhibited the ability of *F. graminearum* to infect maize seeds, with a concentration of 64 μg/mL of B1 completely preventing infection (Figure 6A). Furthermore, in maize seeds treated with bacillomycin D, no detectable synthesis of the two toxins was observed (Figures 6B, C), which further supports the potential of bacillomycin D as a biocontrol agent in mitigating mycotoxin contamination.

### 3.8 SEM and TEM

**Figure 7:**
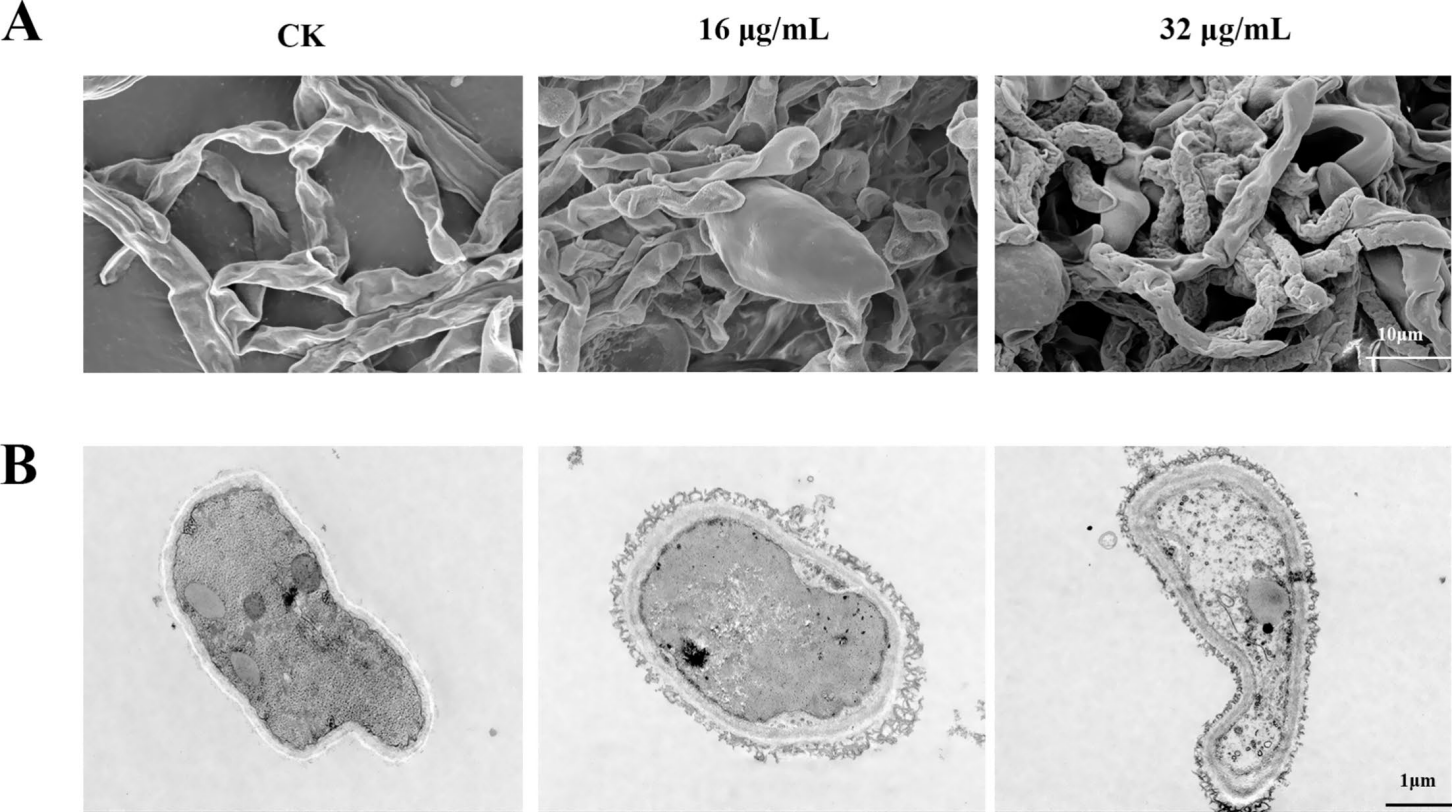
Observation of the microscopic morphology of *F. graminearum* hyphae under treatment with bacillomycin D. **(A)** SEM, **(B)** TEM. CK represents the control group without the use of bacillomycin D treatment.

To investigate the mode of action of bacillomycin D against *F. graminearum*, we examined the microscopic morphology of hyphae following bacillomycin D treatment. SEM analysis revealed that bacillomycin D treatment induced abnormal development in *F. graminearum* hyphae, characterized by vesicular structures and wrinkled surfaces. Moreover, hyphal growth and branching were significantly inhibited (Figure 7A). TEM observations demonstrated significant morphological and structural changes in fungal hyphae treated with bacillomycin D compared to the control group, which exhibited normal hyphal cell structure. Following bacillomycin D treatment, the cell membrane and wall exhibited damage or delamination, the cytoplasm contracted, and the mitochondria displayed abnormal shapes (Figure 7B).

### 3.9 Cell membrane permeability assay

**Figure 8:**
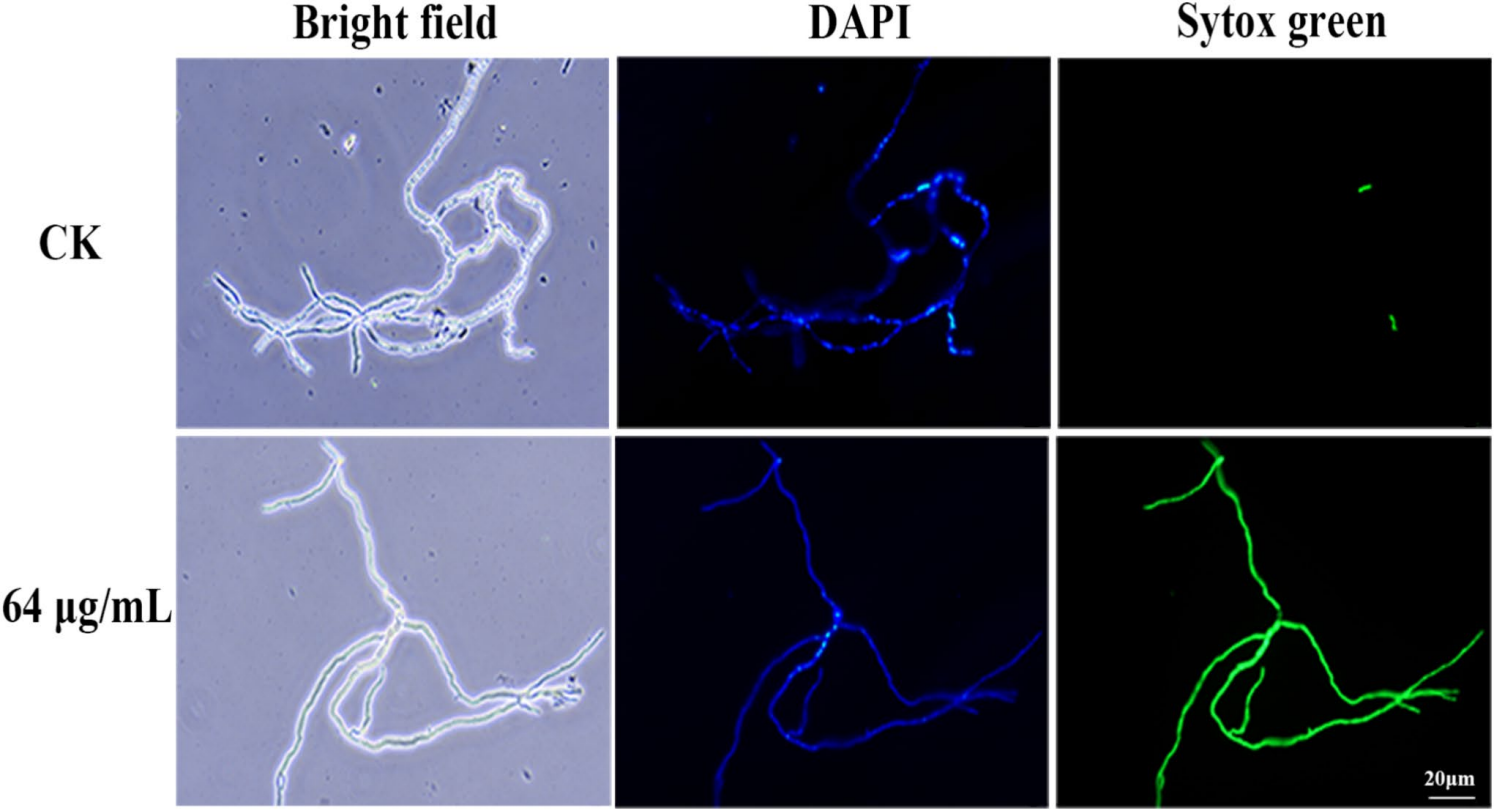
Effect of bacillomycin D on the membrane permeability of mycelium in *F. graminearum*, as demonstrated by Sytox Green staining. 6.67% (vol/vol) methanol served as the control (CK).

To assess the impact of bacillomycin D on membrane permeability, Sytox Green staining was employed. As illustrated in Figure 8, hyphae in the control group displayed weak fluorescence, with some cells remaining unstained by Sytox Green. In marked contrast, hyphae treated with bacillomycin D exhibited intense fluorescence, with the majority of cells stained by the dye. This observation indicates that bacillomycin Ds significantly enhanced cell membrane permeability.

### 3.10 Reactive oxygen species (ROS) and membrane potential detection

**Figure 9:**
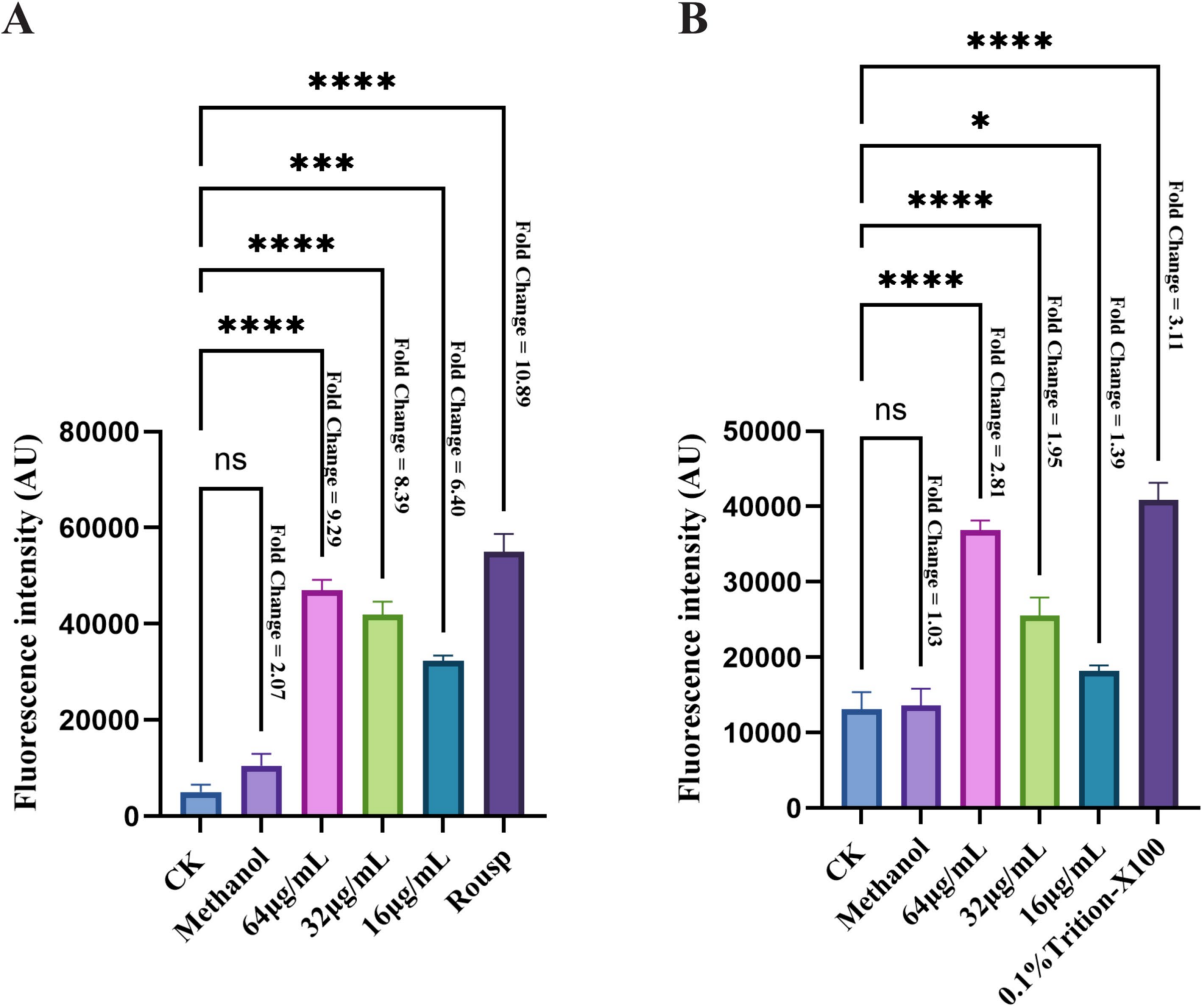
The bactericidal mechanism of bacillomycin D against *F. graminearum*. **(A)** The intracellular ROS content of *F. graminearum* treated with different concentrations of bacillomycin D was detected. **(B)** The membrane potential of *F. graminearum* cells treated with different concentrations of bacillomycin D was detected. CK represents the control group without the use of bacillomycin D treatment. The fold change in fluorescence values relative to the control group for each treatment group is marked to the right of the bar chart. *Significant differences were observed compared to the control group (*p* < 0.05). ***Extremely significant differences were observed compared to the control group (*p* < 0.001). ****Super significant differences were observed compared to the control group (*p* < 0.0001).

The determination of ROS levels in *F. graminearum* cells showed that, compared to the control group, the fluorescence intensity significantly increased after bacillomycin D treatment. This increase exhibited a dose-dependent pattern, indicating that bacillomycin D treatment induced abnormal ROS accumulation (Figure 9A). This suggests that bacillomycin D-triggered ROS generation is a key factor in cell death. Measurements of the membrane potential indicated that bacillomycin D induced depolarization of the *F. graminearum* cell membrane (Figure 9B). These findings suggest that bacillomycin D may exert their bactericidal effects by inducing ROS accumulation, causing damage to the cell membrane.

## 4 Discussion

*Fusarium graminearum* poses significant threats to human and animal health due to its contamination of crops and its ability to produce highly toxic mycotoxins, such as DON and ZEN (Yang et al., 2020). In this study, a strain of Bacillus was isolated from farmland soil, exhibiting significant antagonistic activity against *F. graminearum*. Identification confirmed it as *B. amyloliquefaciens*, whose genome contains a large number of secondary metabolic product biosynthesis gene clusters, indicating its strong potential for biocontrol. A class of cyclic lipopeptides was isolated from its fermentation supernatant, and subsequent analysis revealed it to be a lipopeptide from the iturin family. The inhibitory effects and stability of bacillomycin D against *F. graminearum* were comprehensively evaluated, and its mechanism of action was thoroughly investigated.

Whole genome analysis plays a crucial role in the detection of bacterial secondary metabolites and serves as a powerful tool for discovering novel antimicrobial substances (Wang C. et al., 2023). In this study, the genome of *B. amyloliquefaciens* is 4,038,328 base pairs in size with a G + C content of 46.34%. It encodes a total of 3,837 coding genes, with a total gene length of 3,594,972 bp. The genome of *B. amyloliquefaciens* 4-9-2 is predicted to contain 12 biosynthetic gene clusters. Among these, macrolactin H, bacillaene, fengycin, difficidin, bacillibactin, and bacilysin exhibit 100% similarity to known gene clusters, while surfactin, myxovirescin A1, and Butirosin A/butirosin B show similarities of 78, 21, and 7%, respectively. These secondary metabolites have been demonstrated to possess antibacterial, antifungal, iron-chelating, and biofilm-forming activities (Dat et al., 2021). Furthermore, the genome of *B. amyloliquefaciens* 4-9-2 harbors three unknown biosynthetic gene clusters, highlighting its significant potential as a novel biocontrol agent.

Lipopeptide antibiotics synthesized by *Bacillus* spp. are important antifungal substances, among which iturin exhibits significant inhibitory effects on various fungi (Yaraguppi et al., 2023). The iturin family is diverse, including members such as iturin A, iturin C, iturin D, iturin E, iturin F, iturin W, bacillomycin D, bacillomycin F, bacillomycin L, and mixirins (Wan et al., 2022; Zhou et al., 2020). In this study, a bioactive lipopeptide was obtained from the CFS of *B. amyloliquefaciens* 4-9-2. Based on molecular weight comparison analysis, this lipopeptide belongs to the iturin family and is identified as C_15_-bacillomycin D (Gu et al., 2017; Koumoutsi et al., 2004). Previous studies have reported the effectiveness of iturin family lipopeptides in combating *F. graminearum*. It has been found that iturin A completely kills *F. graminearum* conidia at a minimum inhibitory concentration (MIC) of 50 μg/mL (Gong et al., 2015). Other studies have also shown that bacillomycin D has an inhibitory effect on *F. graminearum*, with a half-inhibitory concentration (IC_50_) of approximately 30 μg/mL (Gu et al., 2017). However, the purification of a single lipopeptide from bacterial metabolites is challenging, and previous studies seem to have noted this as well. For example, in the study on bacillomycin D antagonizing *F. graminearum*, they used a mixture of bacillomycin D with different fatty acid chain lengths (Gu et al., 2017). Researchers have discovered that the hydrophobic chain length of lipopeptides has a non-linear relationship with antifungal activity (Greber et al., 2017). In this study, C_15_-bacillomycin D was purified from the supernatant of *B. amyloliquefaciens* 4-9-2, and its antagonistic effect on *F. graminearum* was found to be similar to reported results in the literature, with an MIC of 64 μg/mL and a IC_50_ of 26.1 μg/mL. These results provide data support for more refined studies on the antagonistic ability of iturin family lipopeptides against *F. graminearum* and experimental support for designing new biocontrol agents based on existing structures in the future.

In fact, the extraction or storage conditions we selected, although commonly reported in literature, may have a potential impact on the antifungal activity of lipopeptides. For example, studies comparing the ethyl acetate extraction method with the acid precipitation method found that the former extracted more types of lipopeptides (such as Kurstakin, Iturin, etc.) and had stronger antifungal activity, suggesting that the acid precipitation method may selectively lose some active components (Dimkić et al., 2017). Other research has found that the Iturin family is more stable to acid, while Surfactin may be more susceptible to pH (Barale et al., 2022; Romero et al., 2007). This also seems to hint at why the lipopeptides we ultimately discovered belong to the Iturin family. We realize that this extraction condition (acid precipitation protocol) may have caused us to miss some potentially antimicrobial substances. This has prompted us to make more attempts in our subsequent research to discover more valuable antifungal active substances from the metabolites of *B. amyloliquefaciens* 4-9-2.

The influence of environmental factors on the antifungal activity of lipopeptides is a topic worth discussing. Studies have reported that lipopeptides form different nanostructures under varying pH conditions, directly affecting their interaction with fungal cell membranes (Xie et al., 2022). Additionally, pH can influence the secondary structure of lipopeptides by modulating their charge state (such as protonation of amino and carboxyl groups), which may also lead to differences in antifungal activity (Ghosh and Fernández, 2020). Most lipopeptides remain stable within the pH range of 5–9, with the optimal range for many being pH 6–8. For example, the AF3 lipopeptide produced by *Bacillus subtilis* has a minimum inhibitory concentration (MIC) against fungi of 4–8 mg/L at pH 6–8 and remains stable after heat treatment and enzymatic digestion (Ramesh et al., 2024). Under extreme pH conditions, the activity and stability of lipopeptides sharply decrease. For instance, the lipopeptides from *Bacillus licheniformis* retain only 60% residual activity at pH 12 and drop to 20% at pH 14 (Chen et al., 2017). This decline may be related to charge neutralization or conformational dissociation, preventing effective binding to fungal cell membranes (Lousa et al., 2020). Of course, there are differences in pH response among different lipopeptide families. For example, studies show that Iturin-type lipopeptides (such as Bacillomycin D) exhibit the strongest activity in neutral to weakly alkaline environments, with the hydrophobicity of their β-amino fatty acid chains reaching optimal balance at pH 7–8, enhancing membrane permeability (An et al., 2024). Fengycins, on the other hand, show higher activity under weakly acidic conditions (pH 5–6), possibly due to increased exposure of fungal cell wall components like glucan and chitin in acidic environments (Tran et al., 2022). Moreover the closed-ring structure of cyclic lipopeptides provides a natural stability mechanism, enhancing their stability and resistance to protease hydrolysis through increased steric hindrance and potential non-covalent interactions (Zhao J. et al., 2022). Some studies have shown that divalent metal ions can form complexes with lipopeptides, thereby enhancing their interaction with lipid membranes and increasing their antimicrobial activity (Ding et al., 2023). These findings align with our research, which shows that bacillomycin D isolated from the metabolites of *B. amyloliquefaciens* 4-9-2 maintains good antifungal activity across a pH range of 2–12, with a significant increase in antifungal activity between pH 8–10. Moreover, its antifungal activity remains stable after heat treatment and enzymatic digestion. Additionally, we found that the antibacterial activity of eumelanin against *F. graminearum* can be significantly enhanced by some divalent cations (such as Fe^2+^, Mg^2+^, Ca^2+^).

Corn is one of the most important sources of food and feed worldwide. In fact, *F. graminearum* contamination in corn crops is very severe, and controlling corn ear rot or stalk rot caused by *F. graminearum* has always been difficult (Adeniji and Babalola, 2018). This is mainly due to the characteristics of the pathogen and the prevailing climatic conditions (Reed et al., 2022). Although biocontrol strategies have been tested in recent decades and have been proven effective in controlling symptoms and mycotoxin production, no final commercial products are currently available on the market. This study demonstrated that the growth of *F. graminearum* on corn seeds was significantly inhibited by the addition of bacillomycin D. As a result, the production of DON and ZEN was substantially reduced. These findings align with the work of Sultana et al., who observed that increasing the concentration of kikar and neem leaves in stored grains led to a rapid decrease in aflatoxin levels (Sultana et al., 2015). Similarly, this study highlights the ability of bacillomycin D to suppress mold growth and inhibit the production of ZEN and DON. Our results are consistent with those of Qian et al., who reported that lipopeptides significantly inhibited the growth of *Aspergillus ochraceus* and the production of ochratoxin A (OTA) in food samples (Qian et al., 2015). These findings underscore the potential of bacillomycin D as effective biocontrol agents for reducing mycotoxin contamination in food and feed.

Lipopeptides may exert antifungal effects through various pathways. *Fusarium oxysporum* has been reported to have its cell membrane integrity disrupted and ROS significantly increased when treated with iturin A at 25 μg/mL or 50 μg/mL (Hua et al., 2023). Additionally, studies have found that surfactin can induce intracellular ROS accumulation in *F. graminearum* cells and demonstrated that this intracellular accumulation of ROS is a key mediator of apoptosis (Liang et al., 2023). Our research complements this and attempts to propose the possibility of multiple antibacterial pathways of iturin. In our study, significant intracellular ROS accumulation was observed in *F. graminearum* after bacillomycin D treatment, accompanied by changes in membrane permeability and a loss of cell membrane potential. Studies reported that ROS accumulation can lead to increased membrane permeability, causing the loss of intracellular substances and further exacerbating cell damage, including loss of membrane potential and ion leakage (Wang B. et al., 2023). For example, lipopeptides such as daptomycin, through calcium-dependent membrane insertion, form cation-selective pores, directly causing membrane depolarization (Patel et al., 2015). These results all suggest that inducing ROS accumulation might be a key mechanism by which lipopeptides exert their bactericidal activity. Of course, we cannot ignore the membrane-disrupting effect of lipopeptides themselves, which are amphipathic molecules composed of hydrophobic lipid chains and hydrophilic peptide head groups, a property that determines their penetration and disruption effects on fungal membranes (Malina and Shai, 2005). These findings suggest that bacillomycin D may inhibit *F. graminearum* through multiple mechanisms, including ROS accumulation and regulation of cell membrane potential. These results provide valuable insights into understanding the antifungal mechanisms of lipopeptide substances.

## 5 Conclusion

In summary, this study demonstrates that *B. amyloliquefaciens* 4-9-2 exhibits robust antagonistic activity against *F. graminearum*. Through whole-genome sequencing, we identified 12 biosynthetic gene clusters (BGCs) in *B. amyloliquefaciens* 4-9-2, highlighting its genetic potential to produce diverse bioactive compounds, including bacillomycin D, which effectively inhibit spore germination and mycelial growth. Notably, bacillomycin D exhibited remarkable stability under extreme conditions, including high temperatures, pH variations, protease exposure, and metal ion interactions. Biological control assays further confirmed the efficacy of bacillomycin D in preventing *F. graminearum* infection and significantly reducing the production of mycotoxins, such as DON and ZEN, in corn. Microscopic observations revealed that bacillomycin D disrupt fungal cell wall and membrane integrity, while further analyses demonstrated that bacillomycin D induce ROS accumulation and loss of membrane potential, ultimately leading to hyphal cell death. These findings underscore the potential of *B. amyloliquefaciens* strain 4-9-2 and bacillomycin D as a promising biocontrol agent for managing *F. graminearum* infections, mitigating mycotoxin contamination, and enhancing food safety.

## Data Availability

The datasets presented in this study can be found in online repositories. The names of the repository/repositories and accession number(s) can be found at: https://www.ncbi.nlm.nih.gov/genbank/, CP175553.

## Footnotes

^1^ http://rast.nmpdr.org/
